# A Historic Review of the Role of CD4^+^ T-Cell Subsets in Development of the Immune Responses against Cutaneous and Visceral Leishmaniases

**DOI:** 10.52547/ibj.26.2.99

**Published:** 2022-01-25

**Authors:** Mohammad Hossein Alimohmmadian, Soheila Ajdary, Fariborz Bahrami

**Affiliations:** Department of Immunology, Pasteur Institute of Iran, 69 Pasteur Avenue, Tehran 13169-43551, Iran

**Keywords:** Immunity, Leishmaniasis, T-Lymphocytes

## Abstract

The heterogeneity of CD4^+^ T cells has been investigated since the late 1970s, when their Th1 and Th2 subsets were coined. Later studies on the cutaneous form of the *Leishmaniasis* were focused on the experimental models of *Leishmania major* infection using the susceptible BALB/c and the resistant C57BL/6 mice. At the early 21^st^ century, the T_reg_ subpopulation was introduced and its role in concomitant immunity, responsible for lifelong resistance of the host to the reinfection was proposed. Subsequent studies, mainly focused on the visceral form of the infection pointed to the role of IL-17, produced by Th17 subset of CD4^+^ T cells that along the neutrophils were shown to have important yet equivocal functions in protection against or exacerbation of the infection. Altogether, the current knowledge indicates that the above four subsets could orchestrate the immune, the regulatory and the inflammatory responses of the host against different forms of leishmaniases.

## INTRODUCTION

Leishmaniases are neglected intracellular protozoan diseases due to infections by different species of *Leishmania* genus in various parts of the world. Based on the official WHO reports, millions of people in the developing countries are at risk of the infection^[^^1^^]^. Amongst the 30 species of *Leishmania* genus recognized today, 20 of them are responsible for the development of distinct clinical manifestations in humans^[^^2^^]^, which cause a broad spectrum of clinical stages of the infection, observed in various parts of the world. They range from asymptomatic infections, small self-healing cutaneous sores, disseminated cutaneous, the disfiguring mucocutaneous, and the fatal visceral forms^[^^3^^]^. Herein, we focus on the role of CD4^+^ T-cell subsets in the development of the immune responses with respect to two prevalent forms of the disease, namely CL and VL. In the Old World, CL is caused by *Leishmania (L.) major*,* L. tropica*, and *L. aethiopica *species and is endemic around the edge of deserts, arid, and semi-arid areas of North Africa, Central Asia, and the Middle East. CL in the New World is mostly caused by *L. braziliansis* and *L. amazonensis *species and is endemic in Central and South Americas. VL is endemic in both the Old and the New World regions. In the Old World, VL is caused by *L. donovani* complex, including *L. donovani donovani* and *L. donovani infantum*. In the New World, VL is caused by *L. chagasi*^[^^3^^,^^4^^]^. In general, leishmaniases are transmitted to humans by the bites of infected female sand flies of *phlebotomus *(in Old World) and *Lutzomia *(in New World) genera, resulting in parasite inoculation into the skin epidermis or the upper layer of the dermis^[^^5^^]^. Macrophages are the primary target of *Leishmania* parasites. The promastigote (i.e. with a flagellum) form of the parasites are internalized by the macrophage phagosomes, forming a parasitophorous after fusion of phagosomes with the macrophage lysosomes where an acidic milieu and numerous proteolytic enzymes are present. Inside the macrophages, *Leishmania* promastigotes differentiate into a resistant and rounded form without the flagellum, named amastigote. The amastigotes can tolerate the hostile environment inside the macrophages and have the ability to survive and proliferate inside the parasitophorous vacuoles. Replication of amastigotes inside the invaded macrophages results in the disruption of the macrophages and invasion of the released amastigotes to the surrounding uninfected macrophages forming lesions (i.e. CL) or the internal tissues and organs (i.e. VL)^[^^6^^]^. 

A bulk of evidence has shown that the outcome of *Leishmania* infections depends upon the challenge between the parasite, as an invader, and the ensued counteracting immune responses by the host, as a defender. Studies have pointed to the important role of T-cell subsets in immunity against CL. Two main subsets of T-cells, namely the CD4^+^ and the CD8^+^ T-cells, were initially perceived to be responsible for mediating immunity against *Leishmania* parasites. However, due to the intracellular nature of the parasitic invasion, the response developed by CD4^+^ T-cell subsets against leishmaniases was historically considered as the main mode of defense. The origin of this perception was a proposed dichotomy, based on murine model studies in which the differentiation of CD4^+^ T-cells towards Th1 functional phenotype results in protection and resistance against the reinfection. Meanwhile, the domination of Th2 response leads to the exacerbation of the disease^[^^7^^,^^8^^]^. However, later findings emphasized a mixed Th responses in humans as well as the involvement of other subsets of CD4^+^ T-cells, namely T_reg_ and Th17 cells^[^^9^^,^^10^^]^, which together contribute to the resistance and the susceptibility against leishmaniases

**The historical milestones**

***T-cell subsets***

Since the early 1970s, questions have arisen regarding the distinct functions of thymus-derived T-cells in the literature. Extensive experiments performed later helped to characterize whether different T-helper and T-cytotoxic functions were directed by the same T-cell^[^^11^^]^. The subsequent experiments using murine models were focused on the identification of thymus-derived T-cell subsets according to their surface markers, namely Ly-1 (CD4), which could mediate helper function and Ly-2 (CD8) with cytotoxic or suppressor functions^[^^12^^,^^13^^]^. Experiments carried out later on leishmaniases, at the end of the 1970's and in the 1980’s, showed the susceptibility of BALB/c mice to uncontrolled *L. major* infection and their failure to indicate delayed-type hypersensitivity to leishmanial antigens^[^^14^^]^. This behavior was suggested to be due to the suppression of the cell-mediated immunity and expansion of the suppressor T-cells^[^^15^^,^^16^^]^. Attempts were also focused on the characterization and functional properties of murine T-cell subsets, specific to *L. major* parasites. In 1982, Louis *et al.*^[^^17^^]^ reported the *in vitro* generation of T-cell subsets and their functional analyses in leishmaniases. In 1983, Dialynas and colleagues^[^^18^^]^ identified a murine T-cell surface molecule, named L3T4, which was induced by the helper/inducer (CD4^+^) T-cell subset. A similar study, performed by Haregewoin and Louis^[^^19^^]^ in 1983 on functional analysis of T-cell subsets specific to *Mycobacterium leprae*, identified Thy-1^+ ^T cells, which showed consistency with Lyt-1^+^ (CD4^+^-T) cell subset. 

***Discovery of four major CD4*** ^+^ ***T-cell subsets***

The results observed on the different multiple functions of helper CD4^+^ T cells indicated the possibility of the existence of distinct subpopulations of CD4^+^ T cells, demonstrating the distinct features of the immune responses. In 1986, Mosmann and co-workers^[^^20^^]^ suggested the existence of two functional subsets of murine CD4^+^ T cells with profound distinct functions and different cytokine profiles. This finding was a crucial progress toward understanding the mechanisms involved in the development of the host immune responses. Later investigations on experimental leishmaniases proved the important role of T-cell subsets in mediating the host immune responses toward either a protective response or the exacerbation of the lesion. According to this dichotomy, inbred mice infected with *L. major* display two distinct clinical and immunological patterns. C57BL/6 or CBA mice are resistant against *L. major *infection and are capable of controlling the infection by producing cytokines such as IFN-, IL-2, and IL-12 (i.e., the Th1 profile). In contrast, severe metastatic infections are developed in susceptible mice such as BALB/c strain in which the infection is disseminated to other organs including the viscera, while their T-cells produce IL-4, IL-5, and IL-13 cytokines (i.e., the Th2 profile)^[^^21^^]^. These two main subsets of CD4^+^ T cells were remained established until the end of the twentieth century. At the turn of the new century (2000 and 2001), another subset of CD4^+^ T cells, named T_reg_, was documented in the domain of autoimmunity by identifying Foxp3 transcription factor^[^^22^^,^^23^^]^ and its unique expression in T_reg_ cells^[^^24^^,^^25^^]^. In 2002, Belkaid and co-workers^[^^26^^]^ suggested that CD25^+^CD4^+^ T_reg_ cells can control *L. major* infection and favor the persistence of a small number of parasites within the skin of the healed host, which develops a concomitant immunity in the host. Later in 2005, the fourth subset was described by Harrington and associates^[^^27^^]^ who published the evidence for the development of a distinct lineage of CD4^+^ effector T cells that could produce IL-17. They named this subset Th17 cells and showed that the development of Th-17 cells is via unique signaling pathways that are independent of those needed for the development of Th1 and Th2 subsets.

**Immune responses and leishmaniases**

***Immune responses against L. major infection in murine models***

Due to the ethical issues associated with human experiments, most of the knowledge regarding leishmaniases had to be acquired through animal models, especially the murine models of the infections. As hinted earlier, the preliminary experiments have shown that the inoculation of *L. major *strains into different inbred mice results in two distinct profiles. While C57BL/6, C3H/He, or CBA inbred mice displayed resistance against *L. major* infection, other inbred mice including BALB/c indicated profound susceptibility to *L. major* infection. Resistant mice could control the infection, and only a small lesion developed at the inoculation site, which would heal spontaneously within two or three weeks. The healing process in the resistant mice is associated with the development of a strong cellular immune response, characterized by the expansion of CD4^+^ T cells producing IFN-, which could then stimulate macrophages to kill the intracellular parasites^[^^28^^]^. The inflammatory cytokines, including IFN-, IL-12, TNF, and transcription factors, such as T-bet and STAT4, were considered to be involved in the generation of a Th1 response. Meanwhile, genetic ablation of these factors results in susceptibility to *L. major* infection^[^^8^^]^. Moreover, cultivation of mononuclear cells of the resistant mice from the peripheral blood or other organs such as LNs in the presence of leishmanial antigen causes the expansion of CD4^+^ T cells, which were documented to produce relatively the high levels of IFN- and the low levels of IL-4 or IL-5, indicating the development a functional Th1 response^[^^8^^]^. Although IFN- was considered as the main cytokine of Th1 response, which could trigger the development of Th1 cells, IL-12 was also deemed to be required to initiate and maintain Th1 cell response against *L. major* infection^[^^29^^]^. Experiments in the 1990s indicated that IL-12 could act directly both on Th1 cells and their precursors or indirectly via inducing IFN- production by T cells and NK cells^[^^30^^,^^31^^]^. In our own experiments, by the analyses of cytokine genes in draining LN of C57BL/6 mice infected with different strains of *L. major*, we also noticed the higher expressions of both *Ifng* mRNA and *Il12* mRNA and the lower levels of *Il4* and *Il10* expressions at five and eight weeks post infection^[^^32^^]^. In contrast, the inoculation of BALB/c mice with *L. major *was shown to lead to a progressive lesion, which gradually enlarged and disseminated to the neighboring organs. A metastatic infection then appeared, which could gradually spread to the visceral organs, while subsequently, the mice became anemic and cachectic with features similar to human VL. The exacerbation of the infection in BALB/c mice was considered to be correlated with defective cellular immune responses and the development of functional Th2 response^[^^16^^]^. Other reports have suggested that the cultivation of PBMCs or LN cells in the presence of leishmanial antigen induced the development of CD4^+^ T cells, which could rapidly produce the high levels of IL-4 and IL-5 with low levels of IFN-, which then assumed as hallmarks of a dominant Th2 response^[^^33^^-^^35^^]^. Moreover, rapid expression of *Il4* mRNA in draining LN of BALB/c mice infected with *L. major* was reported to be produced by Vβ4^+^-Vα8CD4^+^ T-cells^[^^36^^]^. In our experiments, we demonstrated a burst of *Il4* and *Il10* mRNA expression in the draining LN of BALB/c mice infected with four different *L. major *strains at a very early period (three hours), post inoculation. Moreover, different strains of *L. major *were shown to have different impacts on the mammalian immune responses. Indeed, some strains of *L. major *elicited higher levels of IFN- and a lower amount of IL-4 within two months post infection in BALB/c mice, while under similar conditions, other *L. major *strains exhibited lower ratios of IFN-/IL-4 in the LN cells culture^[^^37^^]^. We also obtained similar results concerning the patterns of cytokine mRNA expression in LN of BALB/c mice during early (3-40 hours) and late (1-8 weeks) stages of the infection. Moreover, we showed the influence of different strains of *L. major* in the development of a tendency toward Th1 or Th2 immune responses evaluated by varied cytokine mRNA expressions in BALB/c mice^[^^38^^]^. With respect to the New World leishmaniases, comparable studies on the induction of different cytokine expression patterns by different strains were also reported. For instance, in a study using *L. braziliensis *strains, inoculation of BALB/c mice by a strain indicated a higher ratio of IFN- /IL-10 than another strain, which was measured in their LN cells, at 3 and 15 days after the infection^[^^39^^]^. 

***Immune responses in human CL***

Among the well-studied causative agents of CL in the Old World is *L. major*. The infection caused by this pathogen is manifested in different clinical forms, ranging from a small self-healing papule or nodule to disseminated chronic and non-healing lesions^[^^40^^]^. However, in most cases, it is represented as a single skin lesion that is healed spontaneously within one to nine months^[^^41^^]^. The healing process of CL patients has been considered to be associated with the development of cell-mediated immunity and resistance to the re-infection. Several studies mostly conducted on CL due to *Leishmania* species in the New World have helped to elucidate the important role of T-cell subsets in immunity against human CL in vaccinated individuals and patients with natural *Leishmania* infections. These experiments indicated that the cultured PBMCs of vaccinated subjects against *L. braziliensis* antigens as well as CD8^+^ T-cell populations and CD4^+^ T cells in patients with active *L. braziliensis* infection are expanded^[^^42^^]^. In another study, in which PBMCs from patients with CL in Brazil were evaluated before and after leishmaniasis treatments, patients with active lesions were reported to have a high proportion of CD4^+^ T cells before the therapy; however, after their treatment and cure, the expansion of *Leishmania*-reactive CD8^+^ T cells was observed^[^^43^^]^. The decreased CD4^+^ and increased CD8^+ ^ T-cells trends was also observed during the healing process of CL caused by *L. braziliensis*^[44]^. With respect to CL in Iran, it has been demonstrated that the upregulation of *Leishmania*-specific Th1-like subsets could mainly be found in individuals recovered from CL or among patients with mild infections, which would lead to protection against the reinfection in most individuals^[^^45^^]^. Our own previous studies on PBMC culture showed that in patients with active lesions and those who have recovered from *L. major *infection, a Th1-like response persists against specific antigens of either promastigote^[^^46^^]^ or amastigote^[^^47^^]^. However, in non-healed cases of CL, the induction of T cells by leishmanial antigen caused the development of a high level of IL-4 as well as low levels of IFN-, which represent a Th2-like pattern^[^^48^^]^. Moreover, we were able to show that the establishment of Th1 response could be observed in individuals with a history of leishmaniasis and also in individuals vaccinated with crude promastigote antigens^[^^49^^]^. In CL caused by *L. major*, we also documented that the outcome of the disease depends on the development of Th1-like response, mainly through the production of IFN- by PBMCs in response to leishmanial antigen, as evidenced by studies in which either crude proteins^[^^50^^]^, or recombinant protein originated from *L. major *amastigotes were used^[^^51^^]^. Aside from the *L. major* infection, we also showed the immune responses elicited in anthroponotic CL caused by *L. tropica* in a study in which patients with acute lesion exhibited Th1-like response; however, in non-healed patients, the production of high levels of IFN-, IL-5, and IL-13 suggested mixed Th1/Th2 responses^[^^52^^]^. Our results obtained from individuals with a history of *L. major* infection suggested that CD4^+^ T cells are the dominant subset of T cells in human CL cases^[^^53^^]^. Altogether, the obtained data pointed to the fact that the outcome of infection and the clinical manifestations of CL in humans are greatly influenced by the expansion of specific T-cell subsets and the type of immune responses that develop. As recently reviewed^[^^54^^]^, IL-27 is another cytokine of importance that can affect the innate and the adaptive immunity with respect to leishmaniases. This cytokine is secreted by dendritic cells and macrophages at the early phase of the infection. Interestingly, it is understood that IL-27 has diverse T-cell-dependent functions relying on the confronting *Leishmania* species and the stage of the infection. For instance, it has a positive role in protection against CL by *L. major* infection by inhibiting IL-4-mediated Th2 response, while it plays suppressive roles against Th1 responses caused by *L. donovani*, *L. infantum*, *L. amazonensis*, and *L. braziliensis* infections. In addition, during the late stages of the infection, IL-27 along with IL-10 is considered to limit tissue damage caused by *L. braziliensis*^[^^54^^,^^55^^]^.

**The newer subsets of CD4** ^+^ **T cells**

The descriptions of new subsets of T cells, namely the T_reg_ in 2002^[^^26^^]^ and Th17 cells in 2005, opened new perspectives for the immunologists in the field of leishmaniases^[^^27^^,^^56^^]^. With respect to T_reg_, it was then suggested to divide them into two categories: First, the natural or nTreg (Foxp3^+^ CD4^+^ CD25^high^) that is derived from the thymus, and second, the inducible T_reg_ or iT_reg_ that is developed in the periphery. One of the major roles of T_reg_ was assumed to counteract the inflammatory immune responses to limit their excessive tissue damage at the sites of inflammation^[^^57^^]^. T_reg_ and Th17 were widely accepted as new subsets of CD4^+^ T cells at the beginning of the 21^st^ century. T_reg_ was then characterized by different surface markers, including CD25^+ ^(IL-2 receptor α chain), GITR, CTLA-4, CD103, and Foxp3 transcriptional factor, and it was evaluated that almost 10-15% of CD4^+^ T cells contained CD25 markers^[^^58^^]^. Although the initial reports specified the function of these subsets to autoimmunity, it was later elucidated that these subsets can influence the outcome of immunity and substantially affect the fate of leishmaniases^[^^27^^]^. It took a decade to have a more clear idea about Th17 subset of T cells, which their *in vitro* development requires IL-6 and TGF-β and their *in vivo* functions need IL-23^[^^59^^]^. The important roles of T_reg_ and Th17 subsets of CD4^+^ T cells in leishmaniases will be discussed below in more details.

**T**_reg_ **in leishmaniases**

Early in the 21^st^ century, the critical roles of T_reg_ in eliciting resistance to leishmaniases were highlighted in several reports. In the self-healing form of the disease in murine models (C57BL/6 or CBA) and also in humans, the protective immune responses against leishmaniasis were expected to be mediated by Th1 response via the production of IFN- by CD4^+^ T cells and IL-12 by dendritic cells. The continuation of this status was perceived to result in the development of a sterile immunity in the host. However, based on animal studies in which mice were infected with *L. major*, the control of CL could not be associated with the complete elimination of the parasites in the host and establishment of sterile immunity. It was then theorized that while CD4^+^ T cells produce cytokines to establish a protective response in the host, a portion of these cells, namely the CD4^+^CD25^+^T_reg_, suppresses the excessive activation of the effector CD4^+ ^T cells, which results in the inhibition of killing of the remaining parasites. Eventually, this feature gives rise to the maintenance of a few parasites in the body, followed by the induction of resistance against the reinfection in host and the development of a concomitant immunity^[^^26^^,^^60^^]^. Later, it was found out that Foxp3 is highly expressed in localized CL lesions of patients infected with *L. guyanensis*, which manifests in unresponsiveness to the treatment^[^^61^^]^. In addition, another study has suggested that T cells actively migrate to the skin lesion of CL patients, infected with *L. viannia braziliensis*, while these cells express phenotypic markers of T_reg_ cells. It has also been demonstrated that T_reg_ can significantly suppress phytohemagglutinin-induced proliferative T-cell responses and may contribute to the functional control of effecter T cell^[^^62^^]^. Other reports emphasized that *Leishmania*-specific nT_reg_ was restricted to the infection sites, and parasite persistence was understood to be required for their survival^[^^63^^]^. A role for natural T_reg_ in a chronic form of *L. major* infection was then suggested by detecting its increased gene expression and staining of nT_reg_ markers^[^^64^^]^. Furthermore, in one of our studies, we observed higher levels of Foxp3 mRNA expression in PBMCs of the asymptomatic population of CL compared to the individuals with healed CL lesions. These results may point to the existence of a population of memory T_reg _in the asymptomatic population^[^^65^^]^.

***Contradicting roles of Th17 cells in leishmaniases***

Initial reports investigating on the role of Th17 in CL and mucosal leishmaniasis due to L. braziliensis in 2009 suggested the enhancement of IL-17 production by PBMCs in the course of the infection and also the production of IL‐17 in the lesions of the patients^[^^66^^]^*.* Using cDNA array technology, higher mRNA expressions of Th17 and T_reg_ markers in the lesion tissues of *L. tropica*-infected patients, compared to a healthy control group, had also been reported^[^^67^^]^. Moreover, the function of Th17 cells in the lesions of patients with mucosal leishmaniasis and in association with infiltration of neutrophils in tissue injury areas were documented^[^^68^^]^. By the first decade of the 21^st^ century, two opposite roles for Th17 were started to be documented. These observations suggested the involvement of IL-17 in pathogenesis due to inflammatory reactions during CL and mucosal leishmaniases. Using the susceptible BALB/c mouse model, it had been shown that during the *L. major* infection, both CD4^+^ T-cells and neutrophils are capable of secreting increased amounts of IL-17 compared to the similar cell types from the resistant C57BL/6 mice. The lesion sizes in *L. major*-infected IL17-deﬁcient mice were signiﬁcantly smaller and had a lower parasite load compared to wild-type BALB/c mice^[^^69^^]^. Hence, a direct relation between elevated IL-17 expression and CL pathology could clearly be observed in the animal models^[^^70^^]^. Also, in the New World, enhanced levels of IL-17 production in CL patients due to *L. braziliensis* were reported^[^^66^^]^; however, in culture supernatants of PBMC obtained from *L. braziliensis*-infected CL patients, higher levels of IL-17 could be observed. Interestingly, such elevated levels of IL-17 were not detectable in the healed CL patients^[^^71^^]^. On the contrary, a protective role for IL-17 was identified for the VL patients whose Th17 cells were considered to be involved with host protective immune response by the secretion of IL-17 cytokines and neutrophil recruitment to the infection sites^[^^72^^]^, detectable by the high serum level of IL-17 in patients who were resistant to the infection or recovered quickly after *L. donovani* infection^[^^73^^]^. *L. donovani* is reported to stimulate the differentiation of Th17 subpopulation to produce IL-17, IL-22, and IFN-. Both IL-17 and IL-22 cytokines are associated with inflammations while having complementary roles in protection against VL. Defects in production of Th17 are reported to lead to an elevated risk of VL^[^^74^^]^. Moreover, the involvement of IL-17 in the pathogenesis of PKDL is proposed and the enhanced expression of Th17 response during PKDL is suggested to be involved in the clearance of the parasite^[^^75^^]^.

**VL and the immune responses**

*L. donovani *and *L. infantum *(and *L. chagasi*, their homologous counterpart in the New Word)^[^^76^^]^ are the main species responsible for causing VL. Infections by these species in human result in two distinct features in terms of clinical manifestations and the immune responses. The majority (80-90%) of the infected individuals develop the subclinical or asymptomatic infection with strong cellular immunity and mostly exhibit positive LST result, lymphocyte proliferation, and development of Th1 response against the parasite^[^^77^^,^^78^^]^. However, in a few percentages of the infected population, the dissemination of the infection from the inoculation sites to visceral organs, including liver, spleen, LN, and bone marrow, via the infected macrophages results in severe clinical manifestations, which are fatal, if left untreated^[^^79^^]^. It has been known for long that VL is characterized by depressed cell-mediated immunity, which could be monitored by an indicator of DTH reaction, such as LST^[^^81^^]^. Also, during the acute phase of VL, LST remains negative. However, LST results become positive for the majority of the cured individuals or in cases of recovered patients from VL^[^^81^^,^^82^^]^. In a study in endemic areas of Meshkin-Shahr (Iran), 70.2% (47/67) of the healed patients from *L. infantum* infection showed positive LST response when tested with leishmanin antigen, prepared from *L. major* species^[^^50^^]^. Although PBMCs from individuals with asymptomatic VL infection induce IL-2, IFN- , and IL-12 in response to stimulation with leishmanial antigens, some data supports the inability of PBMCs from active VL patients to proliferate or respond to leishmanial antigens, due to the lack of induction of IFN- production^[^^83^^-^^85^^]^. As described by Singh and colleagues^[^^86^^]^ in India, whole blood cells from patients with active VL (80%) and healed VL patients (85 %) produce the elevated levels of IFN- . At the time, since the healed individuals showed positive LST response and were resistant to the re-infection, these findings did not support a severe defect in Th1 response in human VL and indicated that the application of an *in vitro* whole blood assay could determine cell-mediated immune responses in VL patients^[^^87^^,^^88^^]^.

***Cytokine responses against VL***

One of the key cytokines used for discrimination of the individuals with asymptomatic *L. donovani* or *L. infantum* infections from patients with symptomatic or severe VL was IFN- . Elevated levels of IFN- as well as IL-12p40, IL-18, and IL-15 have been reported in plasma of patients with symptomatic active VL, compared to asymptomatic and healthy individuals residing in the endemic areas of South-Western Ethiopia^[^^89^^]^. In an earlier survey, the simultaneous increase of IFN- and IL-10 at both plasmatic and intracytoplasmatic levels have been documented in active VL patients. That study also showed that the secretion of inflammatory cytokines, especially IL-8, IFN-, TNF-and IL-6, have major roles in changing the clinical status of active VL patients. Also, a major role for IL-10 in inhibiting the macrophage activation, associated with the active VL disease, was suggested^[^^90^^]^. However, later data from the New World (Brazil) reflected mixed Th1 and Th2 responses in active VL cases, observed by the induction of higher levels of IFN-γ, TNF-α, IL-4, and IL-10 in plasma of the patients, compared to the asymptomatic and the healed individuals^[^^91^^]^. Reports obtained since 2011 from the whole blood assessment of cytokines from VL cases in Bihar (India) changed the former hypotheses regarding the impaired production of *Leishmania*-specific IFN-  by VL patients^[^^86^^,^^87^^]^. Another VL study from Bihar has indicated that CD4^+^ T cells are the main source of IFN-  induction in whole blood cultures that are stimulated with *Leishmania* antigens. It has also been observed that IFN-  produced in active VL patients limits the growth of the parasites^[^^92^^]^. When the capacity of whole blood cells obtained from patients with active VL from Gondar in Ethiopia was compared with the previously-mentioned Bihar data, it was explored that unlike the Bihar results, 

VL patients in Gondar had low or negligible levels of IFN-γ and IL-10; however, their impaired capacity to produce IFN-γ (but not IL-10) was restored in a timely manner, after successful treatments^[^^93^^]^.

In conclusion, the accumulated knowledge so far indicates that the aforementioned four major subsets of CD4^+^ T-cells are involved in the immune responses against different forms of leishmaniases. Many factors including the protozoan pathogen, the sand fly vector, the reservoir and the complexity of the human immune system affect the outcome of leishmaniases. Therefore, using murine models and conventional methods restricted to evaluations of a few indicators seem inadequate to draw a complete picture of the role of CD4^+^ T-cell subsets with respect to the infections. New holistic methodologies in the future, such as transcriptomic approaches, which are capable of evaluating the expression of many genes in human populations at different clinical stages of the disease are expected to advance our understanding in this regard.

## DECLARATIONS

### Ethical statement

Not applicable.

### Data availability

Not applicable.

### Author contributions

MHA conceived the review, wrote and revised the manuscript. SA and FB added to the content and revised the manuscript.

### Conflict of interest

None declared.

### Funding/support

No funding or support was received for this review.
